# Risk Factors of Sleep Disturbance in Older Adults With Dementia: An Actigraphy-Based Validation Study

**DOI:** 10.1093/geroni/igab046.2467

**Published:** 2021-12-17

**Authors:** Jinhee Shin, Eunhee Cho, Bada Kang, Sujin Kim, Sinwoo Hwang, Eunji Kwon, Seok-Jae Heo

**Affiliations:** 1 Yonsei University College of Nursing, Seoul, Seoul-t'ukpyolsi, Republic of Korea; 2 Mo-Im Kim Nursing Research Institute, Yonsei University College of Nursing, Seoul, Seoul-t'ukpyolsi, Republic of Korea; 3 Yonsei University, Seoul, Seoul-t'ukpyolsi, Republic of Korea; 4 Korea Armed Forces Nursing Academy, Daejeon, Taejon-jikhalsi, Republic of Korea; 5 Yonsei University Graduate School, Seoul, Seoul-t'ukpyolsi, Republic of Korea

## Abstract

Sleep disturbance is a common and significant symptom experienced by older adults with dementia. Early detection and timely treatment of sleep disturbance are critical to prevent adverse consequences including decreased quality of life for persons with dementia and increased caregiver burden. While direct observations and sleep diaries are often unreliable, actigraphy is a cost-effective method in measuring sleep problems in older adults with dementia and provides reliable and rich sleep data. Therefore, this study aimed to examine sleep disturbance objectively measured by actigraphy and its risk factors in community-dwelling older adults with dementia in Korea. This is a prospective study consisting of a two-wave dataset. The model was fitted using Wave 1 data (n=151) and then validated using Wave 2 data (n=59). Independent variables were demographics, cognitive and physical function, depressive symptoms, physical activity level, and neuropsychiatric symptoms measured by Neuropsychiatric Inventory(NPI), and clinical factors including dementia type, sedative use, and comorbidities. Sleep disturbance was defined as less than six nighttime sleep hours and sleep efficacy less than 75%. Using the Youden’s Index, the sample was dichotomized into sleep disturbance group (n=83) and sound sleep group (n=68). The results of the generalized linear mixed model showed that the risk factors for sleep disturbance included vascular dementia, age, step count, and having three neuropsychiatric symptoms (i.e., delusions, depression, and disinhibition). Individuals with dementia at risk for sleep disturbance should be identified to prioritize early prevention strategies and individualized interventions. Particularly, management of delusion, depression, disinhibition is critical in preventing disturbed sleep.

